# Long-Term Epidemiological Trends and Regional Disparities in Male Infertility in Central Asia (1991–2023)

**DOI:** 10.3390/ijerph23040466

**Published:** 2026-04-06

**Authors:** Mukhamediyar Mukhamejan, Timur Shamshudinov, Mirzakarim Alchinbayev, Nariman Tabynbayev, Murat Dursun, Assiya Kussainova, Laura Kassym, Oxana Tsigengagel, Nurbolat Zhambylov, Yuliya Semenova

**Affiliations:** 1 Department of Urology and Andrology, Kazakh National Medical University Named After S.D. Asfendiyarov, Almaty 050054, Kazakhstan; muhamedzhan.m@kaznmu.kz (M.M.); tabynbayev_n@kaznmu.kz (N.T.); zhambylov.n@kaznmu.kz (N.Z.); 2 Medical and Diagnostic Center of Otorhinolaryngology, Almaty 050026, Kazakhstan; 3 Department of Urology (Istanbul Medical Faculty), Istanbul University, Istanbul 3445, Turkey; murat.dursun@istanbul.edu.tr; 4 Department of General Medical Practice with a Course of Evidence-Based Medicine, NJSC “Astana Medical University”, Astana 010000, Kazakhstan; kussainova.as@amu.kz (A.K.); kassym.l@amu.kz (L.K.); 5 Department of Epidemiology and Biostatistics, NJSC “Astana Medical University”, Astana 010000, Kazakhstan; ziegenhagel.o@amu.kz; 6 School of Medicine, Nazarbayev University, Astana 010000, Kazakhstan; yuliya.semenova@nu.edu.kz

**Keywords:** male infertility, sociobehavioral determinants, alcohol use, drug use, STIs, Central Asia

## Abstract

**Highlights:**

**Public health relevance—How does this work relate to a public health issue?**

- Provides the first comprehensive comparative analysis of male infertility trends across all five Central Asian countries via standardized GBD 2023 data (1991–2023).
- Addresses male reproductive health within the context of declining fertility and limited policy attention in the region.

**Public health significance—Why is this work of significance to public health?**

- Identifies divergent country-specific trends and age shifts in peak burden, highlighting demographic and epidemiological heterogeneity within Central Asia.
- Aims to demonstrate associations between male infertility burden and selected modifiable sociobehavioral risk factors at the population level.

**Public health implications—What are the key implications or messages for practitioners, policy makers and/or researchers in public health?**

- Suggests integration of male reproductive health into national surveillance and prevention strategies.
- Provides evidence to inform targeted, country-specific reproductive health policies and future analytical research in the region.

**Abstract:**

Background: Male infertility (MI) has become an important public health concern in Central Asia (CA), where declining fertility coincides with limited integration of male reproductive health into national strategies. This study analyzed trends in the prevalence and burden of MI in CA countries and assessed associations with selected modifiable behavioral risk factors. Methods: A longitudinal analysis of Global Burden of Disease (GBD) 2023 data was conducted to estimate the age-standardized prevalence and incidence of MI among men aged 15–49 years in five CA countries from 1990 to 2023. Trends were examined, and fixed-effects panel regression models were applied to evaluate associations between MI burden and alcohol use, drug use, and sexually transmitted infections (excluding HIV). Results: From 1991 to 2023, the age-standardized prevalence increased in four CA countries, whereas Tajikistan showed a decline; the incidence rose across all countries. The peak incidence was most commonly observed at ages 35–39 years, although Tajikistan shifted toward a younger age group, and regression analyses revealed heterogeneous country-specific associations with sociobehavioral risk factors. Conclusions: MI represents a significant demographic and public health challenge in CA, highlighting the need to strengthen surveillance, standardize diagnostics, improve access to care, and integrate male reproductive health into national prevention strategies.

## 1. Introduction

In recent decades, male infertility (MI) has evolved from a narrowly defined clinical condition into a broader medical, social, and demographic challenge within public health systems [1]. According to the most recent guidance from the World Health Organization (WHO), MI is defined as the inability to achieve pregnancy in a female partner after 12 months of regular unprotected intercourse, attributable to disturbances in spermatogenesis or hormonal regulation [2]. Global meta-analytical evidence indicates that infertility affects approximately one in five to one in six couples of reproductive age worldwide, with male-related factors identified in approximately 40–50% of cases, either as an isolated cause or in combination with female factors [3,4]. The highest detection rates of MI are typically observed among men aged 30–39 years [5]; however, decreases in sperm concentration and motility have increasingly been reported even among younger age groups [6].

The etiopathogenesis of MI is multifactorial and encompasses genetic abnormalities, endocrine dysregulation, inflammatory processes, and obstructive conditions affecting the reproductive tract [7]. In recent years, increasing attention has been given to modifiable sociobehavioral determinants that may contribute to impaired male reproductive function [3]. Alcohol consumption and drug use disorders have been associated with reduced testosterone levels, disrupted spermatogenesis, and a greater proportion of morphologically abnormal spermatozoa [8]. The prevalence of sexually transmitted infections (STIs) has increased in the context of multiple sexual partners and inconsistent use of barrier contraception, potentially leading to chronic inflammation of the reproductive tract and obstructive azoospermia [9]. In low- and middle-income countries, additional influences such as labor migration, rapid urbanization, psychosocial stress, and shifts in marital and reproductive patterns further shape the epidemiology of MI [10].

The issue of MI is of particular relevance in the countries of Central Asia (CA), including Kazakhstan, Uzbekistan, Kyrgyzstan, Turkmenistan, and Tajikistan, where historically high fertility has been socially and culturally expected [11]. However, demographic trends over the past two decades indicate a shift in this pattern, with total fertility rates declining across all countries in the region between 2004 and 2024 [12]. A reduction in fertility levels occurring alongside a potential increase in the male contribution to infertility creates an unfavorable demographic context. At the same time, dedicated governmental programs focused on the prevention and early detection of MI remain largely absent in CA, whereas existing reproductive health initiatives primarily emphasize maternal and child health. Consequently, male reproductive health continues to be insufficiently integrated into national public health strategies, underscoring the importance of population-based analyses grounded in comparable international data.

A comprehensive assessment of the global burden of infertility has been conducted within the framework of the Global Burden of Disease (GBD) Study, which provides standardized and comparable estimates of prevalence and years lived with disability for more than 200 conditions across 204 countries since 1990 [13]. Although several publications have examined trends in East Asia, the Middle East, and Europe, CA countries are seldom analyzed as independent regions and are more often incorporated into broader post-Soviet or Eurasian groupings. This aggregation limits the identification of region-specific patterns and constrains the development of targeted policy responses.

Accordingly, the primary aim of this study was to describe and compare long-term trends in the prevalence and burden of MI across CA countries from 1991–2023 via GBD data. The secondary aims included examining age-specific patterns and exploring potential population-level associations with selected behavioral risk factors without implying causal relationships.

## 2. Materials and Methods

### 2.1. Study Design

This study was a longitudinal analysis based on secondary data obtained from the GBD 2023 Study [13]. We applied panel data modeling to evaluate the associations between selected behavioral and infectious risk factors (drug use, alcohol use, and STIs) and the outcome indicator across five CA countries over time.

### 2.2. Data Sources

Data for this study were obtained from the GBD 2023 Study, a comprehensive and systematically curated database that provides global, regional, and national estimates for 375 diseases and injuries and 88 risk factors across 204 countries and territories worldwide [13]. The analysis focused on Kazakhstan, Kyrgyzstan, Tajikistan, Turkmenistan, and Uzbekistan, a group of CA countries that share common historical, socioeconomic, demographic, and healthcare system characteristics, including a legacy of the Soviet healthcare model, comparable population structures, transitional health systems, and similar patterns of environmental and occupational exposures [14]. Data on the age-standardized prevalence of MI (GBD cause ID: D0099.01) and the incidence of the composite category ‘Urinary diseases and male infertility’ (GBD cause ID: 594) MI among men aged 15–49 years in these countries, including estimates of prevalence and incidence for the period 1991–2023, were retrieved from the GBD Results Tool, accessible at https://vizhub.healthdata.org/gbd-results, (accessed on 12 February 2026). This platform is maintained by the Institute for Health Metrics and Evaluation (IHME) at the University of Washington, Washington, WA, USA [13]. Cases of MI were identified via standardized diagnostic classifications based on the International Classification of Diseases, including the International Classification of Disease (ICD-9) codes (606–606.9, V26.5, V26.52) and the International Classification of Diseases (ICD-10) codes (N46–N46.02, N46.022–N46.12, N46.122–N46.9) [15,16].

For the risk–outcome analysis, the dependent and exposure variables were identified. The dependent variable was the age-standardized prevalence rate (per 100,000 population) of the health outcome of interest (as defined in the Section 3), obtained from GBD 2023. Age standardization was performed using the GBD world standard population to allow cross-country comparability. Three primary explanatory variables were included in the analysis: (i) drug use, defined according to the GBD risk factor framework and encompassing opioid use, cocaine use, amphetamine use, cannabis use, and other illicit drug use; (ii) alcohol use, measured as age-standardized exposure estimates in accordance with the GBD comparative risk assessment methodology; (iii) STIs, excluding human immunodeficiency virus (HIV) infection, including syphilis, chlamydial infection, gonococcal infection, trichomoniasis, genital herpes, and other specified STIs as classified in the GBD database.

### 2.3. Ethics Statement

All the data analyzed in this study were derived from the GBD 2023 Study, which was conducted in compliance with the Declaration of Helsinki and relevant regulatory standards. Since this research was based solely on publicly accessible, aggregated, and nonidentifiable data, it was exempt from institutional ethics committee review in accordance with institutional requirements.

### 2.4. Statistical Analysis

#### 2.4.1. Rate Estimation and Trend Analysis

Age-standardized prevalence and incidence rates (expressed per 100,000 population) were analyzed with corresponding 95% uncertainty intervals (UIs).

#### 2.4.2. Age-Specific Patterns and Peak Analysis

To investigate the demographic distribution of MI, we analyzed prevalence rates across seven five-year age intervals ranging from 15–19 to 45–49 years. For each country, we identified the “peak age group,” defined as the 5-year age interval (e.g., 15–19, 20–24 years) with the highest age-standardized prevalence of male infertility in a given year. We determined this interval both at baseline (1990/91) and at the end of the study period (2023). No additional adjustments for potential confounding factors were made when identifying peak age groups or analyzing age shifts, as the primary aim at this stage was to provide a descriptive overview of demographic trends in the distribution of the burden [O5.1]. By tracking movements between these age brackets, we determined the ‘Direction of Shift’ to assess whether the burden is transitioning toward older or younger cohorts. All estimates were reported with 95% UIs to ensure statistical precision. Finally, we compared the magnitude of these peaks to quantify how the intensity of the infertility burden evolved within the most affected groups over the 33-year study period.

#### 2.4.3. Risk–Outcome Association Analysis

The associations between sociobehavioral determinants and the MI burden were examined via country-level fixed-effects (FE) panel regression models. This approach controlled for unobserved time-invariant country-specific heterogeneity (e.g., cultural reporting norms or baseline healthcare infrastructure). The choice of the fixed-effects model was justified by a Hausman test (*p* < 0.05), indicating its superiority over a random-effects model. To account for unobserved time-specific shocks or trends affecting all countries, time fixed effects (year fixed effects) were also included in the models. To address potential serial correlation and heteroskedasticity commonly found in panel data, cluster-robust standard errors (clustered at the country level) were applied. This approach ensures robust inference by accounting for both heteroskedasticity within countries and arbitrary forms of serial correlation over time within each cluster. Predictors included the prevalence rates of alcohol use disorders, drug use disorders, and STIs excluding HIV. Both the dependent (DALYs) and independent variables were log-transformed; thus, the resulting coefficients (β) were interpreted as elasticities, representing the percentage change in MI DALYs associated with a 1% change in risk factor prevalence. To address potential multicollinearity among risk factors, variance inflation factors (VIFs) were calculated for all independent variables. Variables with VIF values exceeding a threshold of 5 were carefully reviewed for exclusion or transformation to ensure that multicollinearity did not unduly influence the regression coefficient estimates. All variables retained in the final model exhibited acceptable VIF values.

All the statistical analyses were performed via R (version 4.3.1) and Python (version 3.10) via the statsmodels and pwlf libraries. Two-sided *p* values < 0.05 were considered statistically significant.

## 3. Results

Table 1 presents the age-standardized prevalence and incidence rates (per 100,000 population) with 95% UI in 1991 and 2023 across five CA countries, along with the relative percentage change over the study period. Between 1991 and 2023, the prevalence increased in Kazakhstan (+33.2%), Kyrgyzstan (+7.6%), Turkmenistan (+1.9%), and Uzbekistan (+11.5%), whereas a substantial decrease was observed in Tajikistan (−33.8%). In 2023, the highest prevalence rate was reported in Kyrgyzstan (2565.3 per 100,000; 95% UI: 1413.1–4459.9), whereas Tajikistan had the lowest (1164.5; 95% UI: 842.4–1596.3). The incidence rates increased in all five countries during the study period. The largest relative increase was observed in Turkmenistan (+17.4%), followed by Uzbekistan (+14.8%), Kazakhstan (+14.0%), and Tajikistan (+13.0%), whereas Kyrgyzstan presented the smallest increase (+7.1%). In 2023, Kazakhstan had the highest incidence rate (5127.7; 95% UI: 4214.6–6096.0).

Table 2 compares the peak age-specific prevalence of MI in CA countries in 1990/91 and 2023. In 1990/91, the highest prevalence was most commonly observed in the 35–39-year age group (Kyrgyzstan, Tajikistan, Turkmenistan, and Uzbekistan), whereas in Kazakhstan, the peak occurred earlier, in the 25–29-year age group. By 2023, the peak age had shifted to 35–39 years in Kazakhstan, indicating a transition toward older age at maximum prevalence. In contrast, Tajikistan demonstrated a shift toward a younger peak age group (30–34 years). Kyrgyzstan, Turkmenistan, and Uzbekistan maintained the same peak age group (35–39 years) across the study period, with relatively stable prevalence rates. Kazakhstan showed a notable increase in the peak prevalence rate (from 2167.2 to 3299.8 per 100,000), whereas Tajikistan experienced a marked reduction in peak prevalence (from 3051.7 to 1967.3 per 100,000).

Table 3 presents the country-specific elasticity coefficients (β) for estimating the associations between selected behavioral risk factors and the MI DALY prevalence in CA from 1991 to 2023. Considerable heterogeneity was observed across countries. Drug use disorders demonstrated a positive and statistically significant association with MI DALY prevalence in Kazakhstan (β = +1.138; 95% CI +0.850, +1.426) and in the regional fixed-effects model (β = +0.805; 95% CI +0.680, +0.930). Tajikistan had the largest positive coefficient for drug use disorders (β = +4.220; 95% CI −0.500, +8.940), although statistical significance was not indicated. In contrast, negative associations were observed in Kyrgyzstan (β = −1.561; 95% CI −3.800, +0.678) and Uzbekistan (β = −1.160; 95% CI −2.500, +0.180). Alcohol use disorders were negatively associated with MI DALY prevalence in Kazakhstan (β = −0.727; 95% CI −0.950, −0.504) and in the regional model (β = −0.246; 95% CI −0.360, −0.132), both of which were statistically significant. Tajikistan was the only country demonstrating a significant positive association with alcohol use disorders (β = +0.903; 95% CI +0.700, +1.106). The remaining countries had small, nonsignificant negative coefficients. For STIs (excluding HIV), Kazakhstan presented a strong positive association (β = +2.267; 95% CI −0.150, +4.684), although the difference was not statistically significant. Kyrgyzstan and Uzbekistan also had positive but nonsignificant coefficients. In contrast, Tajikistan showed a statistically significant negative association (β = −4.666; 95% CI −7.200, −2.132). At the regional level, the fixed-effects estimate suggested a small positive but nonsignificant association (β = +0.092; 95% CI −0.080, +0.264).

**Figure 1:**
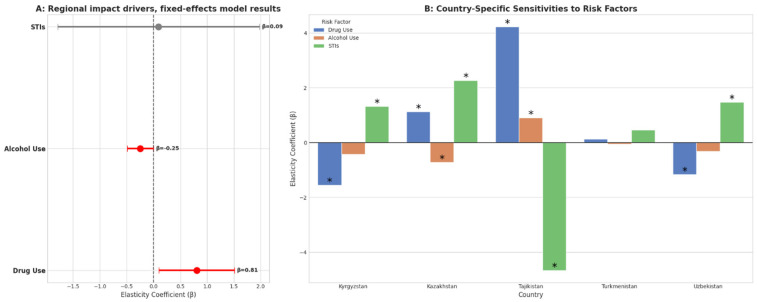
Regional fixed-effects estimates (**A**) (red indicates statistical significance; grey indicates non-significance) and country-specific elasticities of drug use, alcohol use, and STIs (**B**) in Central Asia. Statistical significance is denoted by asterisks (*).

Figure 1A presents the regional fixed-effects model estimates for the associations between selected risk factors and the MI DALYs. At the regional level, drug use demonstrated the strongest positive and statistically significant association (β = 0.81; *p* < 0.05), indicating that increases in drug use were associated with a substantial increase in the outcome indicator MI DALYs. Alcohol use showed a modest negative and statistically significant association (β = −0.25; *p* < 0.05). In contrast, STIs exhibited a small positive but statistically nonsignificant association (β = 0.09).

Figure 1B illustrates country-specific elasticities, highlighting considerable heterogeneity across CA countries. Drug use was positively and statistically significantly associated with the outcome of MI DALYs in Kazakhstan (β = +1.138; *p* < 0.05), with the strongest effect observed in Tajikistan (β > 4, *p* < 0.05). Conversely, negative associations were observed in Kyrgyzstan (β = −1.561) and Uzbekistan (β = −1.160) (both *p* < 0.05), whereas the association in Turkmenistan was negligible (β = +0.128). Alcohol use had mixed effects across countries. A positive and statistically significant association was observed in Tajikistan (β = +0.903; *p* < 0.05), whereas Kazakhstan demonstrated a modest but significant negative association (β = –0.727; *p* < 0.05). In the remaining countries, associations were small and not statistically significant. STIs were positively associated with the outcome in Kyrgyzstan, Kazakhstan, and Uzbekistan (all *p* > 0.05), with Kazakhstan showing the greatest positive elasticity. In contrast, Tajikistan demonstrated a strong negative association (β = –4.666; *p* < 0.05), and Turkmenistan showed a weak, nonsignificant positive effect (β = +0.468).

## 4. Discussion

### 4.1. Regional and Age-Specific Features of Prevalence and Incidence Trends Across Central Asia

The present study demonstrates heterogeneous trends in MI across CA countries over a 32-year period. The increase in prevalence observed in Kazakhstan, Kyrgyzstan, Turkmenistan, and Uzbekistan contrasted with a marked decline in Tajikistan, likely reflecting differences in demographic trajectories, diagnostic accessibility, and the organization of specialized care. For example, the relatively high prevalence reported in Kyrgyzstan in 2023 (2565.3 per 100,000 population) may indicate improved case detection and increased awareness of the contribution of males to infertility [17]. In contrast, the decline observed in Tajikistan may hypothetically be associated with migration-related shifts in population age structure, as well as variations in socioeconomic conditions, healthcare access, and the availability of reproductive health services [18]. According to Shan et al. (2025), in high-income countries, the establishment of assisted reproductive technology centers and sperm banks, along with advances in microsurgical techniques and improvements in varicocele management, has contributed to increased detection and more effective treatment of MI [19]. In contrast, Maqsood et al. (2021) attributed the lower reported prevalence in some settings to differences in data quality, including potential underestimation, underreporting, and the lack of standardized regional registries [20]. Comparable variability in prevalence has been reported in Eastern Europe, East Asia, and western sub-Saharan Africa, as well as in other low- and middle-income settings [4,21].

The increase in incidence across all five countries, ranging from +7.1% in Kyrgyzstan to +17.4% in Turkmenistan, suggests the continued influence of physiological, cultural, and behavioral risk factors [5]. The highest incidence rate recorded in Kazakhstan in 2023 (5127.7 per 100,000 people) may reflect improved detection and broader socioeconomic changes, such as urbanization and a growing burden of metabolic and endocrine disorders [22]. The potential contribution of expanded screening activities should also be considered. Similar patterns have been documented in other post-Soviet countries, such as Russia and Ukraine, where intensified surveillance was accompanied by a temporary rise in reported incidence alongside declining mortality rates [22,23].

The persistence of peak rates in the 35–39-year age group across most countries is consistent with international evidence indicating that MI is most frequently identified during the third and fourth decades of life [3,24]. Several contemporary studies have shown that men older than 35 years, compared with younger individuals, present significantly lower ejaculate volume and reduced total and progressive sperm motility, findings interpreted as age-related changes in spermatogenesis [24,25]. These alterations are thought to be associated with a functional decline in Sertoli cells and impaired supportive activity within the seminiferous tubules, as well as age-related modifications in hormonal regulation, including a reduction in Leydig cell number and gradual decreases in testosterone production [26,27]. The shift in peak prevalence toward older age groups in Kazakhstan may reflect delayed healthcare-seeking behavior, diagnostic practices, and sociocultural factors. For example, Kwizera Tsinda et al. (2025) reported that men typically undergo andrological evaluation only after prolonged unsuccessful attempts to conceive, often exceeding the recommended 12-month period [28]. In addition, in many Asian settings, male sexual dysfunction is frequently underreported or concealed due to patriarchal norms and concerns about social stigma [29]. In contrast, the earlier peak observed in Tajikistan may be associated with patterns of earlier marriage, cultural taboos surrounding sexual health, and lower levels of awareness among younger populations [30].

### 4.2. Impact of Modifiable Sociobehavioral Factors on Male Infertility

Among the sociobehavioral determinants examined at the regional level, drug use disorders demonstrated the most consistent positive association with the prevalence of MI. The high elasticity coefficient observed in Tajikistan may hypothetically reflect country-specific patterns of drug use, potentially shaped by its geographic position along major trafficking routes [31]. In contrast, the negative coefficients identified in Kyrgyzstan and Uzbekistan may be tentatively associated with differences in reporting practices, policy environments, or coverage of addiction services, although these interpretations cannot be confirmed with the available data [32]. A substantial body of clinical and experimental research supports the detrimental effects of illicit drug use on male reproductive parameters, including reduced sperm concentration and motility, increased proportions of morphologically abnormal spermatozoa, elevated DNA fragmentation, and suppression of the hypothalamic–pituitary–gonadal axis, ultimately leading to decreased testosterone production and impaired spermatogenesis [8,33]. In addition, a study by Fonseca et al. (2022) demonstrated that drug use was associated with a significantly lower semen pH (7.86 ± 0.082 vs. 8.09 ± 0.031; *p* = 0.010) and an overall deterioration in sperm quality parameters [34].

Associations with alcohol use disorders were generally weak and negative across most countries in the region; however, a statistically significant positive relationship was identified in Tajikistan. This apparent inconsistency may be explained by potential measurement error, limitations inherent to ecological modeling, and inaccuracies in exposure assessment [35,36]. A meta-analysis by Li et al. (2021), which included 46 studies, reported that even moderate alcohol intake was associated with reduced erectile function, with risk increasing in a nonlinear dose–response manner and rising substantially in cases of chronic abuse [37]. Pathophysiologically, these effects have been linked to alcohol-related hepatic dysfunction resulting in hormonal imbalances, including elevated estrogen and reduced testosterone levels, as well as increased oxidative stress and endothelial dysfunction within the penile vasculature [38].

The observed positive association between STIs and MI prevalence, particularly in Kazakhstan and Kyrgyzstan, may potentially and presumably reflect differences in screening intensity, reporting practices, and underlying behavioral patterns [39]. Bacterial contamination of the ejaculate has been shown to adversely affect male fertility by reducing sperm motility, damaging DNA integrity, and impairing acrosomal function, potentially through apoptosis induced by bacterial antigens and increased oxidative stress mediated by excessive reactive oxygen species production [40]. The statistically significant negative association observed in Tajikistan may be related to the country’s smaller population size, substantial labor migration flows, and more conservative sociocultural norms [18].

In addition to behavioral determinants, MI is a multifactorial condition that encompasses clinical and biological causes not captured in aggregated GBD data. Recent systematic reviews and meta-analyses indicate that varicocele [41], metabolic disorders [42], and smoking [43] are significantly associated with impaired semen parameters and increased sperm DNA fragmentation. Both contemporary and earlier studies have also demonstrated a consistent relationship between elevated body mass index and reduced spermatogenesis, which is mediated by hormonal dysregulation and oxidative stress [44,45]. Evidence from genetic and experimental research further suggests that approximately 10–15% of clinically significant cases are attributable to chromosomal abnormalities and gene mutations [46]. In addition, environmental exposures, such as endocrine-disrupting chemicals, heavy metals, air pollution, and occupational hazards, have been shown to adversely affect male reproductive function and contribute to reduced fertility [47].

Although these findings highlight the importance of modifiable sociobehavioral determinants as potential targets for preventive strategies in CA, it should be emphasized that the associations identified are based on ecological analysis and do not permit direct causal inference.

### 4.3. Implications for Public Health Policy

Differences in health system organization and national policy priorities across CA countries may also contribute to the observed variation in MI indicators. In Kazakhstan, the presence of mandatory social health insurance, expanded healthcare financing, and the inclusion of reproductive services within the primary healthcare package may create more favorable conditions for the detection of infertility cases [48]. In addition, since 2021, a state-funded in vitro fertilization program has been implemented, expanding access to assisted reproductive technologies (ART) [48]. However, this program is focused primarily on infertility treatment rather than on the prevention and early detection of male factor infertility. In Uzbekistan, Kyrgyzstan, Tajikistan, and Turkmenistan, reproductive health issues are formally reflected in regulatory frameworks, but access to specialized care may depend more strongly on resource constraints, the pace and scope of health system reforms, and the quality of monitoring systems [49].

In this context, CA countries would benefit from shifting the emphasis from a predominantly treatment-based model toward a prevention-oriented framework. Potential directions may include strengthening early detection and improving patient referral pathways within primary healthcare, as well as enhancing the integration of male reproductive health into national health programs. In resource-constrained settings, the incorporation of screening for sexually transmitted infections, endocrine disorders, and modifiable risk factors into existing primary care services may represent a pragmatic approach. However, the development of targeted, country-specific strategies requires more granular data, including information at the individual and subnational levels, underscoring the need for further research in this area.

Overall, the region requires an intersectoral approach that combines noncommunicable disease prevention, occupational health measures, health education, and efforts to reduce stigma related to male reproductive disorders. Such a strategy could enhance the effectiveness of demographic and social policy across CA.

### 4.4. Study Limitations

Several methodological considerations should be taken into account when interpreting the findings, as the analysis is based on estimates from the GBD Study. These estimates are model-derived and synthesized from multiple data sources rather than primary clinical registries and may therefore be influenced by data availability, quality, and underlying modeling assumptions.

The study relies on aggregated national-level data and follows an ecological analytical framework. Accordingly, the observed associations reflect population-level patterns and should not be interpreted as causal relationships at the individual level. In addition, potential temporal lags between exposure to risk factors and the development or diagnosis of infertility were not explicitly addressed, which may have affected the observed associations.

The panel analysis was restricted to five CA countries, which may limit the statistical stability of the estimates and constrain their generalizability. Cross-country differences in diagnostic practices, case ascertainment, and access to andrological and reproductive health services could have further contributed to the observed variability.

Sensitivity analyses were conducted through alternative model specifications rather than formal lagged exposure models or the exclusion of outlier years, given the aggregated structure of GBD data and the study’s focus on long-term trends. The key associations remained consistent across these specifications, supporting the robustness of the findings. However, additional sensitivity testing using alternative prevalence indicators was not feasible within the constraints of the GBD dataset. Wider uncertainty intervals, particularly in age-specific analyses, likely reflect data sparsity and regional limitations in primary data collection, which should be considered when interpreting the results.

An important limitation is the absence of key andrological indicators, including semen parameters, hormonal profiles, and other clinical characteristics, as well as detailed information on behavioral and occupational exposures, which limits a more comprehensive assessment of the determinants of male infertility. Finally, the aggregated and model-based nature of the data, together with the ecological study design, does not allow for the development of detailed, country-specific health policy strategies, and the findings should therefore be interpreted with appropriate caution.

Despite these limitations, this study provides a comparable regional overview of the burden of male infertility and offers a basis for future research using individual-level data, longitudinal designs, and qualitative approaches to better understand the underlying mechanisms and barriers to care.

## 5. Conclusions

The present study indicates that MI represents a substantial component of the contemporary demographic and reproductive health agenda in CA countries, warranting systematic attention from health authorities. The findings highlight the need to strengthen surveillance mechanisms, improve diagnostic standardization, and enhance access to specialized services. The incorporation of male reproductive health into national public health strategies and the promotion of evidence-based preventive approaches may contribute to the development of sustainable frameworks aimed at preserving the reproductive potential of the population in the region.

## Figures and Tables

**Table 1:** Absolute and relative measures of male infertility burden, Central Asia, 1991–2023 (aged 15–49 years).

Country	Measure	1991 Rate (95% UI)	2023 Rate (95% UI)	Relative Change (%)
Kazakhstan	Prevalence	1567.1 (1140.6–2166.5)	2087.8 (1181.9–3822.1)	+33.2%
	Incidence ^a^	4498.0 (3806.3–5338.6)	5127.7 (4214.6–6096.0)	+14.0%
Kyrgyzstan	Prevalence	2383.1 (1415.8–3787.8)	2565.3 (1413.1–4459.9)	+7.6%
	Incidence ^a^	4461.8 (3746.7–5283.5)	4778.6 (4013.4–5639.5)	+7.1%
Tajikistan	Prevalence	1759.3 (972.7–2973.5)	1164.5 (842.4–1596.3)	−33.8%
	Incidence ^a^	4188.9 (3520.8–4967.6)	4732.9 (3995.8–5546.7)	+13.0%
Turkmenistan	Prevalence	2106.5 (1165.3–3773.9)	2146.8 (1162.0–3873.1)	+1.9%
	Incidence ^a^	3966.4 (3338.1–4648.1)	4658.0 (3925.8–5466.9)	+17.4%
Uzbekistan	Prevalence	1564.6 (938.3–2554.4)	1744.2 (958.9–3078.1)	+11.5%
	Incidence ^a^	4146.2 (3511.1–4891.2)	4761.2 (3992.2–5627.9)	+14.8%

Note: Rates are per 100,000 male population. UI, uncertainty interval. ^a^ Refers to a composite GBD category of “Urinary diseases and male infertility” (Cause ID: 594).

**Table 2:** Comparison of peak age-specific male infertility incidence in Central Asian countries between 1991 and 2023.

Country	Peak Age Group (1991)	Prevalence Rate per 100,000 (95% UI)	Peak Age Group (2023)	Prevalence Rate per 100,000 (95% UI)
Kazakhstan	25–29	2167.2 (1353.0–3289.9)	35–39	3299.8 (1146.4–7710.0)
Kyrgyzstan	35–39	4418.3 (1604.6–9910.9)	35–39	4417.5 (1606.3–9821.7)
Tajikistan	35–39	3051.7 (1143.7–6994.1)	30–34	1967.3 (1333.5–2729.2)
Turkmenistan	35–39	3621.8 (1224.8–8218.4)	35–39	3549.7 (1229.8–7949.8)
Uzbekistan	35–39	2934.5 (1120.5–6974.3)	35–39	2930.7 (1091.4–6959.7)

Note: 95% UI—95% uncertainty intervals.

**Table 3:** Country-specific elasticity coefficients (β) of behavioral risk factors associated with male infertility DALY prevalence, 1991–2023.

Country	Drug Use Disorders (β) (95% CI)	Alcohol Use Disorders (β) (95% CI)	STIs (Excluding HIV) (β) (95% CI)
Kazakhstan	+1.138 * (+0.850, +1.426)	−0.727 * (−0.950, −0.504)	+2.267 (−0.150, +4.684)
Tajikistan	+4.220 (−0.500, +8.940)	+0.903 * (+0.700, +1.106)	−4.666 * (−7.200, −2.132)
Kyrgyzstan	−1.561 (−3.800, +0.678)	−0.437 (−0.900, +0.026)	+1.323 (−0.500, +3.146)
Uzbekistan	−1.160 (−2.500, +0.180)	−0.320 (−0.750, +0.110)	+1.483 (−0.300, +3.266)
Turkmenistan	+0.128 (−0.300, +0.556)	−0.068 (−0.250, +0.114)	+0.468 (−0.100, +1.036)
Regional (FE)	+0.805 * (+0.680, +0.930)	−0.246 * (−0.360, −0.132)	+0.092 (−0.080, +0.264)

Note: β represents the elasticity coefficient (percentage change in male infertility DALY prevalence per 1% change in risk prevalence). * Statistically significant at *p* < 0.05; FE = fixed effects. 95% CI—95% confidence interval.

## Data Availability

The original data presented in the study are openly available at GitHub 3.5.5 at https://github.com/laurakassym-a11y/Manuscript-Mukhamejan-Dataset.git (accessed on 12 February 2026).

## Abbreviations

The following abbreviations are used in this manuscript:

CA	Central Asia
FE	Country-Level Fixed-Effects
GBD	Global Burden of Disease
HIV	human immunodeficiency virus
IHME	Institute for Health Metrics and Evaluation
MI	male infertility
STIs	sexually transmitted infections
UIs	uncertainty intervals
WHO	World Health Organization
